# Ascending Aortic Aneurysm With Dissection in the Second Trimester of Pregnancy

**DOI:** 10.7759/cureus.31805

**Published:** 2022-11-22

**Authors:** Marcos Sosa, Kunal Patel, Rosa Flores, Bryna Peplinski, James Murray, Terrika Jones, Pedro Reyes

**Affiliations:** 1 Obstetrics and Gynecology, Lakeland Regional Health, Lakeland, USA

**Keywords:** risk factors for aortic aneurysm, symptoms of aortic dissection, chest pain in pregnancy, aortic aneurysm in pregnancy, aortic dissection in pregnancy, ascending aortic aneurysm

## Abstract

Aortic aneurysm in pregnancy is uncommon. A dissecting aortic aneurysm in pregnancy is less common. If present, a dissecting aortic aneurysm can lead to poor outcomes for both the mother and the fetus, including death. Swift recognition of the signs and symptoms of a dissecting aortic aneurysm can prevent a delay in diagnosis and possibly improve maternal-fetal outcomes. The report discusses a case of a pregnant patient who experienced a dissecting aortic aneurysm in the second trimester despite having none of the commonly associated risk factors. The evaluation, diagnosis, and surgical management of dissecting aortic aneurysm in pregnancy are discussed.

## Introduction

An ascending aortic aneurysm (AsAA) is a localized dilation of the first segment of the aorta [1]. The pathophysiology of an AsAA most often involves cystic medial necrosis, histologically presenting as elastic fiber degeneration and smooth muscle cell dropout of the tunica media [2]. AsAA may be asymptomatic until clinical manifestation occurs including chest pain and shortness of breath. Type A aortic dissection includes the ascending aorta up to the point proximal to the brachiocephalic artery and may include a segment of the brachiocephalic trunk. Type B aortic dissection includes the portion of the aorta distal to the brachiocephalic artery. Severe chest pain and shortness of breath may be a sign that an aortic aneurysm has progressed to an aortic dissection [3]. Ultimately, aortic rupture can occur. Untreated acute ascending aortic dissections possess a mortality rate of 1%-2% per hour after onset, presenting a major challenge for hospital personnel with regard to the timing of diagnosis [4]. Similarly, type A aortic dissections surgically treated carry a mortality rate of approximately 20% within the first 24 hours and 50% by one month after clinical presentation [3,4]. Predisposition to thoracic aortic aneurysm (TAA) includes atherosclerosis, blood vessel inflammation, traumatic injury, and genetic conditions such as Marfan syndrome [5].

A recent publication addressing aortic aneurysms in females noted growing evidence indicating that aneurysm formation and behavior may differ based on sex [6]. A trend is noted of thoracic aortic aneurysms (TAA) (including AsAA) being more prevalent in males; however, the outcomes are worse in females [6]. A publication demonstrated that females have a 40% increased risk of mortality in comparison to males and are at three times greater risk of TAA dissection and rupture [7,8].

Although rare, most vascular dissections in pregnancy occur in the ascending aorta [9,10]. A publication estimated an associated AsAA in a pregnant patient followed by a rare, life-threatening complication of aortic dissection to have an incidence of 0.0004% of all pregnancies [11]. Although this number may seem small, there is evidence that up to half of all aortic complications (dissections and ruptures) in females under 40 years of age were associated with pregnancy [9]. More literature is needed regarding AsAA in pregnancy. In this article, we present a rare case of a 30-year-old female who presented with a dissecting aortic aneurysm during the second trimester of pregnancy.

## Case presentation

A 30-year-old Caucasian G1P0 at 25 weeks’ gestation dated by a first-trimester ultrasound presented to the emergency department with a complaint of upper back pain and heartburn that started 24 hours prior. She noted the upper back pain to be worsening over the most recent two hours. In addition, she noted bilateral leg numbness and tingling that began two hours prior to presentation to the emergency department. Her chest pain was 4/10 on the pain scale at the onset, but at the time of presentation, it was described as 8/10. She noted feeling mildly short of breath for the recent two hours. She denied any pregnancy-related concerns. She noted positive fetal movement, denied a loss of amniotic fluid from the vagina, and denied vaginal bleeding. She denied any other past medical history including hypertension, connective tissue disorders, heart disease, or congenital cardiac anomalies. There was no recent trauma to her chest, back, or abdomen. She denied prior thoracic or abdominal surgical procedures.

**Figure 1:**
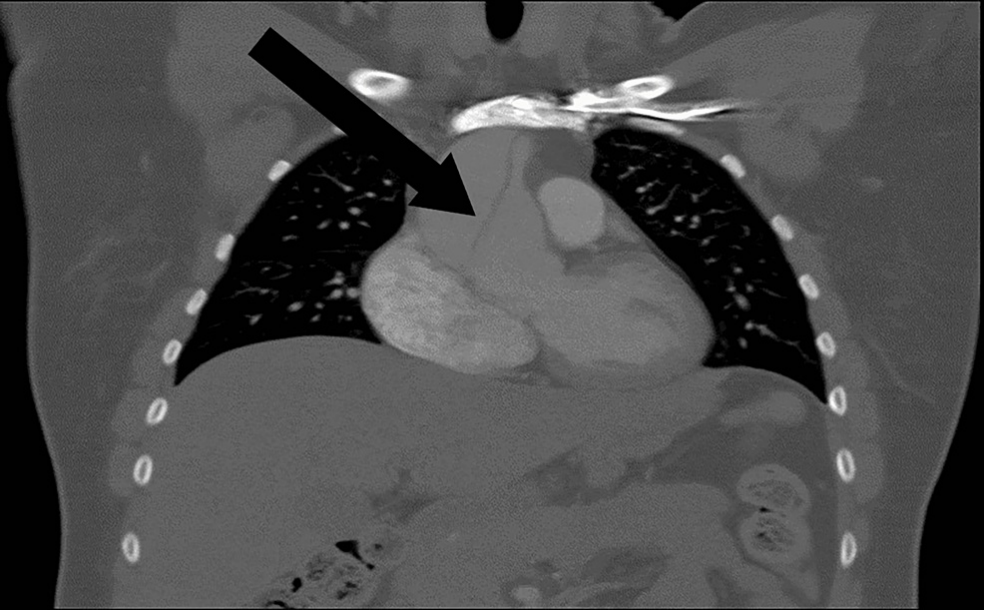
CT coronal image of the ascending aortic aneurysm dissection (black arrow) CT: computed tomography

**Figure 2:**
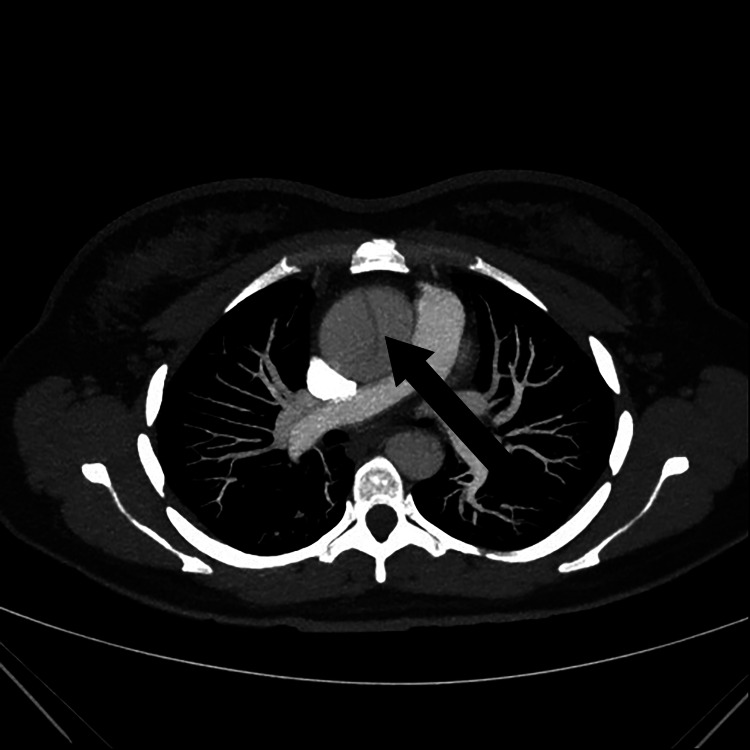
CT axial image of the ascending aortic aneurysm dissection (black arrow) CT: computed tomography

A set of laboratory values were drawn and included a normal hemoglobin of 13 ng/dL, a normal white blood cell count of 12×10^3^/uL, and a normal platelet count of 214×10^3^/uL. A COVID-19 RNA-PCR was negative. Her aspartate aminotransferase (AST) was 24 units/L. Her troponin was <0.019 ng/mL. Telemetry demonstrated sinus tachycardia without evidence of arrhythmia. A brief bedside ultrasound revealed a live fetus with cardiac activity at 148 beats per minute. The emergency room provider had a suspicion of a possible pulmonary embolus and ordered a computed tomography (CT) scan of the chest. A computed tomography scan was performed and revealed a 4.2 cm ascending thoracic aortic aneurysm with dissection. The dissection involved the ascending thoracic aorta through the arch and extending into the descending thoracic aorta and upper abdominal aorta. Images of the dissecting aortic aneurysm can be seen in Figure 1 and Figure 2.

A cardiothoracic surgeon was consulted. An urgent vascular surgical intervention with a Hemiarch repair was recommended. Repair of the aortic dissection would potentially compromise uterine blood flow and therefore lead to possible fetal compromise. Therefore, the obstetrician planned for a concurrent emergency cesarean delivery.

An emergency cesarean delivery was performed. A live neonate weighing 860 g was delivered. Immediate surgical repair of the ascending aortic dissection was done following the cesarean delivery. The repair of the aorta using a 24 mm Hemashield graft was performed and went without complication. She was transferred to the cardiovascular intensive care unit (ICU) for postoperative care. Her postoperative course was uncomplicated, and she was transferred from the ICU to the medical-surgical floor on postoperative day 2. The patient was discharged home on postoperative day 7.

## Discussion

Although the occurrence of aortic pathologies is rare, with an incidence of 0.4 per 100,000 person-years in the female population under the age of 45, when they do occur, they can lead to catastrophic consequences for both the mother and the fetus. During pregnancy, the aortic diameter may increase in size secondary to the changes in maternal cardiac physiology and estrogen concentration. Increased estrogen concentration is known to act on estrogen receptors in the aorta, leading to the degradation of elastin fibers in the tunica media. As the physiologic changes of pregnancy increase the aortic diameter, this can develop into a thoracic aortic aneurysm [12]. Moreover, there is an increase in maternal circulating blood volume, heart rate, and cardiac output. Additionally, hypertensive disorders of pregnancy such as gestational hypertension and preeclampsia may increase the aortic diameter. As the physiologic changes of pregnancy increase the aortic diameter, this can develop into a thoracic aortic aneurysm. Females with a thoracic aortic aneurysm are at increased risk of an acute aortic dissection and the substantial morbidity and mortality associated with the diagnosis. A study found that pregnancy increases the risk of an acute aortic dissection with an odds ratio of 25 [13]. Morbidity and mortality increase without prompt recognition of the dissection. Type A aortic dissections, if unrecognized, have a mortality rate of 20% by 24 hours and 40% by day 7 [13]. Therefore, it is imperative that a prompt and guided evaluation ensues at the hint of an aortic dissection [13]. The complexity of diagnosing an aortic aneurysm is that the patient may not experience any symptoms until an aortic dissection or aortic rupture occurs. Data could not be found on the percentage of patients presenting with a specific symptom that leads to the diagnosis of aortic aneurysm in pregnancy. The preeminent finding in a dissecting aortic aneurysm is chest pain. A study noted that the incidence of chest pain in type A dissecting ascending aortic aneurysm is 83% [14]. Moreover, patients describe the pain associated with a dissecting aortic aneurysm as the “worst-ever pain” experienced in 93% of cases [14].

In the case noted above, the patient presented with chest pain and shortness of breath. The patient also noted a two-hour history of bilateral leg numbness and tingling that is perhaps attributed to altered blood flow to the lower extremities. The initial presentation led to an evaluation for a suspected pulmonary embolism. Other diagnoses in the differential that cause chest pain in pregnancy include gastroesophageal reflux, referred pain from acute cholecystitis, pancreatitis, myocardial infarction, and pneumonia. Her troponin levels and electrocardiogram (EKG) did not demonstrate a cardiac etiology. Resultant imaging demonstrated the aortic dissection, and prompt treatment was pursued.

The patient had no personal or family history of genetic connective tissue disorders including Marfan syndrome, Loeys-Dietz syndrome, Ehlers-Danlos syndrome, or familial thoracic aorta dissection syndrome. Despite this fact, thoracic aortic aneurysms can occur in pregnancy without any risk factor other than pregnancy itself. As noted above, the physiologic changes in pregnancy, namely, increased blood volume, increased cardiac output, increased heart rate, and changes in vessel structure, can lead to an aortic aneurysm and subsequent aortic dissection.

The timing of delivery is critical in the management of pregnant patients with aortic aneurysms and aortic dissection. In females with known aortic aneurysms, the surgical management of aortic root replacement is typically delayed until the third trimester or postpartum in a nonurgent setting. In the third trimester, cesarean delivery and repair of the aortic root can be performed after optimizing for potential neonatal complications including respiratory distress syndrome and neurodevelopmental impairment. Corticosteroids administered to the mother if 33 weeks and six days gestation or less may decrease the risk of respiratory distress syndrome. However, the timing of corticosteroid administration may be different depending on facility guidelines. Magnesium sulfate administered to the mother may decrease the risk of neurodevelopment impairment in the newborn. The timing of magnesium sulfate administration is usually reserved for preterm deliveries, but each facility may have its specific guidelines. Delivery planning should be performed using a multidisciplinary team with the obstetrician, maternal-fetal medicine provider, neonatologist, and cardiothoracic surgeon providing input. In immediate life-threatening settings, emergency cesarean delivery can be performed with concurrent cardiac surgery. Favorable maternal-fetal outcomes have been demonstrated [15]. In the postoperative care of these patients, emphasis is placed on blood pressure management to prevent recurrent aortic dilation; typically, this is accomplished using beta-blockers [16].

## Conclusions

Pregnancy may increase the risk of an aortic aneurysm when compared to the nonpregnant state. Pregnancy itself may be a risk factor for aortic aneurysm and aortic dissection. Prompt recognition and a high index of suspicion are imperative to reducing maternal and fetal morbidity and mortality. Chest pain that is described as severe is the most prominent indicator of a dissecting aortic aneurysm. When evaluating a pregnant patient with a complaint of chest pain, there is a host of diagnoses in the differential. A notation of severe chest pain, or the “worst-ever chest pain,” should lead to immediate evaluation for a dissecting aortic aneurysm. Once diagnosed, a multidisciplinary team is essential for favorable outcomes. The timing of fetal delivery and subsequent aorta repair requires the evaluation of current maternal status with the decision to maximize outcomes for both the patient and the fetus.
